# White matter connectivity linked to novel word learning in children

**DOI:** 10.1007/s00429-024-02857-6

**Published:** 2024-09-26

**Authors:** Clara Ekerdt, Willeke M. Menks, Guillén Fernández, James M. McQueen, Atsuko Takashima, Gabriele Janzen

**Affiliations:** 1 Donders Institute for Brain, Cognition and Behaviour, Radboud University and Radboud University Medical Centre, Nijmegen, the Netherlands; 2 Max Planck Institute for Psycholinguistics, Nijmegen, the Netherlands; 3 Behavioural Science Institute, Radboud University, Nijmegen, the Netherlands

**Keywords:** Word learning, Development, White matter, Structural connectivity

## Abstract

Children and adults are excellent word learners. Increasing evidence suggests that the neural mechanisms that allow us to learn words change with age. In a recent fMRI study from our group, several brain regions exhibited age-related differences when accessing newly learned words in a second language (L2; Takashima et al. Dev Cogn Neurosci 37, 2019). Namely, while the Teen group (aged 14–16 years) activated more left frontal and parietal regions, the Young group (aged 8–10 years) activated right frontal and parietal regions. In the current study we analyzed the structural connectivity data from the aforementioned study, examining the white matter connectivity of the regions that showed age-related functional activation differences. Age group differences in streamline density as well as correlations with L2 word learning success and their interaction were examined. The Teen group showed stronger connectivity than the Young group in the right arcuate fasciculus (AF). Furthermore, white matter connectivity and memory for L2 words across the two age groups correlated in the left AF and the right anterior thalamic radiation (ATR) such that higher connectivity in the left AF and lower connectivity in the right ATR was related to better memory for L2 words. Additionally, connectivity in the area of the right AF that exhibited age-related differences predicted word learning success. The finding that across the two age groups, stronger connectivity is related to better memory for words lends further support to the hypothesis that the prolonged maturation of the prefrontal cortex, here in the form of structural connectivity, plays an important role in the development of memory.

**Supplementary Information:**

The online version contains supplementary material available at 10.1007/s00429-024-02857-6.

## Introduction

Word learning continues across the lifespan and vocabulary is one of the only measures of cognitive ability that does not decline with age (Hartshorne and Germine 2015). While both young children and adults excel at word learning, behavioral studies indicate that there are differences in how the two groups accomplish this task. For example, children perform more poorly in initial word learning than adults but compensate for this with better offline word consolidation (Weighall et al. 2017; James et al. 2018). Furthermore, naps have a different effect on word learning for children compared to adults: while children’s recall of newly learned words improved after a nap, the adults performed more poorly (Van Rijn et al. 2023). There is emerging evidence that there are also age-related differences in how the process of learning new words unfolds at the neural level. It is not yet fully understood, however, which structural connections support word learning at different stages of development, though this is one key piece of the puzzle of the neural mechanisms of word learning. In this study therefore, we investigated age-related differences in white matter structures that play a role in word learning.

To date, numerous neuroimaging studies have established a network of key brain regions involved in novel word learning in adults. These include regions of the language network such as the inferior frontal gyrus (IFG), the middle temporal gyrus (MTG), superior temporal gyrus (STG), inferior parietal gyrus (IPG), and angular gyrus (AG; Mestres-Missé et al. 2008; Friederici 2011; Price 2012; Yang et al. 2015; Nichols and Joanisse 2016; Friederici et al. 2017; Razorenova et al. 2020). While these brain regions have been investigated in adults, less is known about the activation of the language network in developing brains in relation to word learning.

During a recent fMRI study conducted in our laboratory, participants learned words in a language unknown to them (Japanese). This fMRI study forms the starting point for the current study. When retrieving newly learned L2 words, participants in the Young group (8–10 years) activated right hemisphere regions more than the Teen group (14–16 years), whereas the Teen group showed more activation in left hemisphere regions, including the left inferior frontal gyrus (IFG), left inferior parietal lobe and left supplementary motor area (Takashima et al. 2019). In the current study, we complement these functional correlates of word learning in development with the investigation of the white matter connectivity that underlies this ability. By analyzing the diffusion weighted imaging (DWI) data from the same sample, we aim to unravel the relationship between brain structure, especially connectivity, and neural activity during novel word learning in children and adolescents. Specifically, we ask if age-related differences in functional activation are accompanied by similar age-group effects in white matter connectivity and furthermore, we ask if white matter connectivity strength is related to word learning ability.

### Theoretical accounts of brain development applicable to word learning

The two-component framework of episodic memory development proposes that the different maturational trajectories of brain regions can explain the age-related differences seen in memory from childhood into adolescence and adulthood (Shing et al. 2010). While the medial temporal regions mature earlier, the prefrontal cortex (PFC) develops more slowly and is still maturing well into adolescence (Gogtay et al. 2004). Together, the prefrontal cortex and medial temporal regions play an important role in memory. The PFC has been postulated to subserve the strategic component of episodic memory while the medial temporal lobe has been linked to the associative component (Shing et al. 2010). The late maturation of the PFC is thus thought to play an important role in the improvement of episodic memory from childhood to adulthood. That is, the more mature or adult-like the PFC is, the better the memory ability for intentional learning. The recent fMRI results from our laboratory suggest that also in word learning, which relies heavily on memory ability, age-related differences can be observed in the functional recruitment of the PFC: the Teen group recruited the left IFG, part of the PFC, more than the Young group during retrieval of newly learned words (Takashima et al. 2019). In the current study investigating the structural connectivity that supports word learning, we investigated if the white matter connectivity of the regions that differed between age groups in functional activation, including the left IFG, also exhibit age-related differences or are related to L2 word learning.

In the language domain, a pattern that has been observed in many studies is the increasing specialization of the left hemisphere for language (Szaflarski et al. 2006; Enge et al. 2020; Olulade et al. 2020; Gonzalez et al. 2021), although there are studies that have found no differences in lateralization between children and adults (Gaillard et al. 2003; Weiss-Croft and Baldeweg 2015; Ozernov-Palchik et al. 2024). Increasing lateralization of language related processing has been postulated to be related to the improvement of language ability throughout development. For example, in children, lateralization was positively correlated with vocabulary, indicating that leftward lateralization of the arcuate fasciculus was related to a larger vocabulary (Lebel and Beaulieu 2009). Takashima and colleagues (2019) report more activation in the right hemisphere in the Young group compared to the Teen group when processing newly learned L2 words. This is in contrast to the Teen group showing more activation in the left hemisphere. By investigating the structural connectivity that underlies these activation differences in the left and right hemisphere regions, in the current study, we explored if lateralization of the white matter connectivity plays a role in the development of word learning.

Finally, dynamic systems theory (e.g. Hulstijn 2020; Hiver et al. 2022; van Dijk and van Geert 2023) interprets second language development as a complex system of different interacting components with the focus on intra-individual variability. The theory can provide a relevant theoretical context for the current study which links structural and functional brain development to behavioral differences in second language word learning. Especially structural changes like white matter development and the connection to functional and behavioral changes at different stages of development have been mostly disregarded.

### Development of white matter connectivity and the relationship with language learning

In-vivo study of the human brain white matter is made possible by diffusion weighted imaging (DWI), which capitalizes on the white matter’s property of exhibiting restricted water diffusion (Beaulieu 2002). White matter is composed of the axons that connect cortical regions. The microstructure of the white matter, including the strength of connectivity between given regions, has been linked to ability in a wide range of domains including motor skills, literacy, as well as language [for a review on the role of diffusion MRI see Assaf et al. (2019), and how it relates to behavior, Johansen-Berg (2010)]. By studying the strength of connections between brain regions, differences in behavior can be explained. The variation in white matter structure that gives rise to both age-related as well age-independent individual differences can thus be explored with DWI.

Overall, the structural connectivity of the brain’s white matter continues to develop throughout childhood and adolescence. This development peaks in early adulthood, follows different developmental trajectories in different tracts (Giedd et al. 1999; Lebel and Beaulieu 2011; Palmer et al. 2022) and is related to the maturation of the cortex (Cafiero et al. 2019; Corrigan et al. 2021). White matter tracts in the brain can be reconstructed using probabilistic tractography (Behrens et al. 2007). Streamline density is one measure that can be derived from probabilistic tractography and has been proposed to reflect connectivity strength (Behrens et al. 2003). Meaningful differences in connectivity strength between clinical groups (Theisen et al. 2017) as well as between typically developing children and adults (Qi et al. 2019) have been reported. During development, indices reflecting stronger connectivity [e.g. higher fractional anisotropy (FA); higher streamline density] increase with age (Skeide et al. 2015; Qi et al. 2019). Previous studies have combined fMRI and DWI in the same participants to determine the structural connectivity of a functional area (e.g. Neef et al. 2018; Jeon et al. 2019; for a review on studies linking structure and function see Litwińczuk et al. 2023). This approach, which is not often taken in developmental studies, has the benefit of providing additional, and therefore more comprehensive knowledge about the neural underpinnings of studied behavior. In this study we use this method, using functional activation clusters as seeds for tractography, to investigate the connectivity strength of white matter tracts related to L2 word learning in a developmental sample.

In adults, the white matter tracts involved in word learning include the left arcuate fasciculus (AF; Lopez-Barroso et al. 2013) as well as the uncinate and inferior longitudinal fasciculi which have been related to word learning, such that higher myelination was related to better word learning performance (Ripollés et al. 2017). Following a brief period of word learning, changes in white matter microstructure have been measured in adults in the inferior frontal gyrus, middle temporal gyrus, and inferior parietal lobule, and changes in white matter microstructure correlated with learning rate in the middle temporal and supramarginal gyri (Hofstetter et al. 2017). This experience-related plasticity suggests the direct involvement of these white matter areas during word learning. In children, the AF has also been implicated in word learning (François et al. 2016) and vocabulary knowledge (Lebel and Beaulieu 2009; Su et al. 2018). Additionally, the white matter of the middle temporal gyrus has been linked to initial word learning success in children (Ekerdt et al. 2020) and the white matter structure of the right AF in children who could recall more newly learned words after a nap (Van Rijn et al. 2023). These findings suggest that both the dorsal (AF) and ventral (unciate and inferior longitudinal fasciculus) white matter tracts of the language network are implicated in word learning. To date, the structural connectivity related to word learning has not been directly compared between different developmental age groups. Due to the protracted maturation of the white matter, it is possible that different white matter tracts support word learning at different stages of development.

In the current study, we set out to investigate the structure–function relationship in age-related differences in word learning by probing the white matter structures that underlie the brain regions showing age-group differences in functional activity. We asked two questions: first, are there differences in the streamline density of white matter tracts underlying the gray matter regions that differed functionally between the Young and Teen groups (Takashima et al. 2019)? And second, does connectivity strength predict word learning success? We hypothesize that the white matter connectivity (i.e. streamline density) originating from these gray matter brain regions will be stronger for the Teen compared to the Young group due to ongoing white matter maturation during childhood and adolescence (Verhoeven et al. 2010; Lebel et al. 2012). Additionally, we hypothesized a positive correlation between connectivity strength and word learning. Based on the observations from the memory and language literature that the protracted maturation of the PFC and the specialization of the left hemisphere for language play a role in improvement in both of these domains, we were furthermore interested whether this pattern from the fMRI data could be observed in the structural connectivity as well.

## Methods

### Participants

DWI data from 21 children (Young group) between the ages of 8 and 10 years (15 female, 6 male, mean age = 10.08, SD = 0.77) and 23 adolescents (Teen group) between the ages of 14 and 16 years (16 female, 7 male, mean age = 15.77, SD = 0.92) who participated in our group’s previous fMRI study (Takashima et al. 2019) were included in the analysis. All participants were typically-developing, native Dutch-speaking, and right-handed. DWI data were collected from 24 children and 23 adolescents, however, three children were not included in the current analysis due to insufficient data quality caused by movement of the participant in the scanner. All participants and their parents/guardians were informed about the study, gave written consent and were compensated for their participation. The study was approved by the ethical committee of the Faculty of Social Sciences, Radboud University (ECSW2013-0410-134, CMO waiver 45516.091.13).

### fMRI task: lexical decision task

**Fig. 1:**
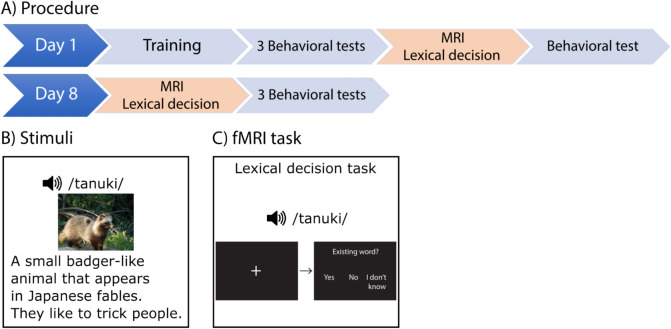
Study overview. On day 1, after training and three behavioral tests assessing knowledge of the newly learned Japanese words, MRI data, including those from a lexical decision task, were collected. Participants then completed a final behavioral test to assess memory for newly learned Japanese words. On day 8, there was no additional training of the words but MRI data, including those from the lexical decision task, were again collected, followed by 3 behavioral assessments of memory for the words that had been learned on day 1. DWI data was collected only during one MRI acquisition; for the majority of participants on day 1. Figure is adapted from Takashima et al. (2019)

All participants aurally learned 30 Japanese nouns depicting objects during a training phase using pictures and Dutch definitions (Fig. 1B). All the objects used were unfamiliar to Dutch adults, as assessed in a pilot study (e.g. a traditional Japanese instrument, a Japanese animal). The training was conducted outside of the scanner and consisted of multiple word learning tasks, beginning with an encoding task, followed by a cued-recall task, a word repetition task, a picture naming task, a free-recall task, and ending with another encoding task. In total, the training session lasted approximately 2 h during which each Japanese word was presented 16 times. Next, participants were tested on their knowledge of the newly learned Japanese words with three behavioral tasks: a free recall, a cued-recall, and a semantic priming task. After these three assessments, participants performed a lexical decision task inside the MRI scanner. Four conditions, each consisting of 30 words, were presented through in-ear headphones in random order: (1) Japanese trained words, (2) Dutch words, (3) Japanese pseudowords, and (4) Dutch pseudowords. Participants had to indicate with a button press (‘yes’, ‘no’, ‘I don’t know’) whether they thought the stimulus was a real word. After one week, participants performed this lexical decision task for the second time inside the MRI scanner without additional training (Fig. 1; for additional details see Takashima et al. 2019). Participants additionally completed tasks of L1 vocabulary (PPVT-NL), digit span, word span and logical processing (Raven’s progressive matrices).

### Image acquisition

MR images were acquired on a Siemens MAGNETOM SKYRA 3 T Scanner (Siemens Healthcare, Erlangen, Germany) using a 32-channel head coil. We acquired 61 whole-brain diffusion-weighted images (*b* = 1000 s/mm^2^) and 8 non-diffusion-weighted images (*b* = 0 s/mm^2^) using a multiplexed echo planar imaging sequence (2.2 mm isotropic resolution; TR = 8100 ms; TE = 90.0 ms; 64 slices; GRAPPA 2; acquisition time = 9 min 45 s). Additionally, we acquired T1- weighted anatomical scans (1 mm isotropic resolution; TR = 2300 ms; TE = 3.03 ms; flip angle 8; FOV 256 × 256 × 192 mm, acquisition time 5 min 21 s). For the details of the functional scan protocol, see Takashima et al. (2019).

### Tractography seed regions

The seed regions for tractography were based on the six functional regions that exhibited age-related activation differences in the lexical decision task (i.e. group differences for the contrast correctly responded Japanese words > Dutch words); these are the left IFG, left IPL, left SMA, right IPL and two clusters in the right MFG (Fig. 3A). These functional ROIs were extracted using the MarsBar tool for SPM (http://marsbar.sourceforge.net/). The ROIs were aligned to the individual participant’s native space and the voxels located within the white matter mask served as seed regions.

### Diffusion weighted imaging preprocessing and tractography

Following visual data inspection, preprocessing included the estimation of the susceptibility-field distortion (Schilling et al. 2020). This field was passed to FSL’s eddy v.6.0.3, a tool that corrects calculated susceptibility-field distortion and combines this with estimating and correcting gross subject movement and eddy current-induced distortions (Andersson and Sotiropoulos 2016). Slices with signal loss caused by subject movement coinciding with the diffusion encoding were detected and replaced by predictions made by a Gaussian Process (Andersson et al. 2016). Additionally, intra-volume subject movement was corrected with eddy’s slice-to-volume motion model and susceptibility-by-movement correction was applied (Andersson et al. 2017, 2018). Changes to the susceptibility-induced distortions caused by subject movement were adjusted (Andersson et al. 2018). Data quality at the single subject level and for comparison at the group level was assessed using the eddy QC tools (Bastiani et al. 2019). The diffusion tensor was fit using FSL’s dtifit to create white matter masks, and the FA maps were used to estimate the transformations to standard space (FSL_HCP1065_FA_1mm). Next, the fiber orientation distribution was determined in each voxel with bedpostX_gpu (Hernandez-Fernandez et al. 2019). Probabilistic tractography was then performed in native diffusion space within the white matter mask using probtrackX_gpu, seeding from the six functional regions that exhibited age-related differences in new L2 word recognition mentioned above. We initiated 5000 streamlines per seed region voxel (curvature threshold = 0.2; step length = 0.5 mm) based on the default settings of probtrackx.

### Statistical analyses: tractography

Tractograms were log transformed, normalized by dividing each voxel by the maximum number of streamlines possible, and warped into standard space (FSL_HCP1065_FA_1mm). For each seed, we took the average of all individual participants’ tractograms, and thresholded this at 0.2 to reduce the number of spurious streamlines. These thresholded group tractograms (visitation maps), served as masks for statistical analyses. As a first step, we tested for group differences between the Young and the Teen group in relative streamline density within the visitation maps originating from the functional seeds. Next, we correlated relative streamline density within the visitation maps with the behavioral variable, L2 word learning success. This was the mean accuracy score from the lexical decision task which participants did in the scanner twice, once on day 1 and once on day 8. Additionally, we conducted an interaction analysis to test for an effect of age group on the relationship between average lexical decision accuracy and relative streamline density. For these analyses we included gender as a covariate and used FSL’s randomise, a tool to conduct nonparametric permutation testing of neuroimaging data (https://fsl.fmrib.ox.ac.uk/fsl/fslwiki/Randomise; Winkler et al. 2014).

All results were corrected for multiple comparisons. The age group comparison and the interaction analyses were corrected with threshold-free cluster enhancement (TFCE; Smith and Nichols 2009). The brain-behavior correlations were corrected for multiple comparisons with a cluster-size correction using AFNI’s (version 16.1.15) 3dFWHMx and 3dClustSim, with a cluster-forming threshold of *p* < 0.001 and cluster size larger than required at the* p* < 0.05 level, Bonferroni-corrected for the number of tracts.

### Regression analyses: L2 word learning success in relation to age and white matter

For the regions of white matter connectivity where age group differences were significant, we performed further analyses with the average extracted streamline density. We asked if the white matter connectivity of those regions could predict L2 word learning success. The analyses were conducted in RStudio, using the ‘step’ function from the stats package for the stepwise regression, and PROCESS for R to conduct the mediation analyses testing the indirect effect of age on L2 word learning success through white matter connectivity.

## Results

### Behavioral results

**Fig. 2:**
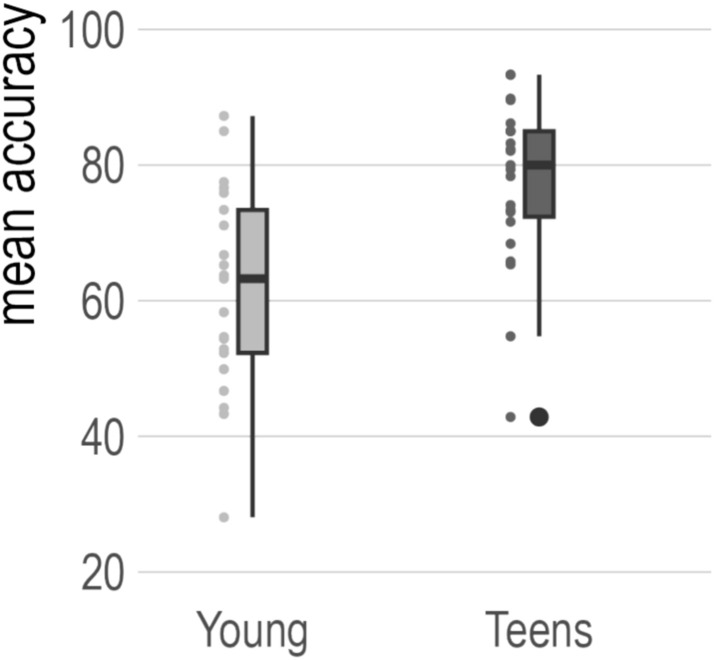
Overview of L2 word learning success behavioral scores. L2 word learning success was defined as the average of the accuracy of the two sessions of lexical decision that participants completed during the scans on day 1 and day 8. The boxplot visualizes the median, 25^th^ and 75^th^ percentile as well as the min and max of the accuracy data. In the Teen group, there is one outlier on the low end, defined as Q1 (25^th^ percentile) −1.5 * IQR. Individual data points are visualized to the left of the respective box plots. *IQR* interquartile range

The behavioral score used in the current analyses was L2 word learning success, the mean accuracy of the two sessions of lexical decision that participants did in the scanner. The Young group performed worse (mean = 61.45%, standard deviation = 15.10%) than the Teen group (mean = 77.47%, standard deviation = 12.24%), see Fig. 2.

The two groups did not differ in L1 vocabulary, digit span or logical reasoning, see Table 1. There was a difference between the groups in the word span measure. This measure does not have a standardized score, and therefore we compared the raw scores which makes the difference expected, with the Teen group showing a higher word span score, on average, than the Young group.

**Table 1:** For all measures besides the logical reasoning test, t-test results are reported. For logical reasoning, we report the results of a Wilcoxon rank sum test because the data did not fulfill the assumptions for a t-test

Behavioral measure	Young group	Teen group	Test statistic	p-value
L1 vocabulary (standard score)	107.29 (11.42)	105.30 (11.68)	t(42) = 0.57	0.57
Digit span (standard score)	11.90 (2.86)	10.70 (2.75)	t(42) = 1.43	0.16
Word span (raw score)	5.95 (1.02)	7.17 (1.47)	t(42) = −3.17	0.003
Logical reasoning (percentile)	79.10 (19.56)	67.61 (28.99)	W = 302.5	0.15

### Tractography results

**Fig. 3:**
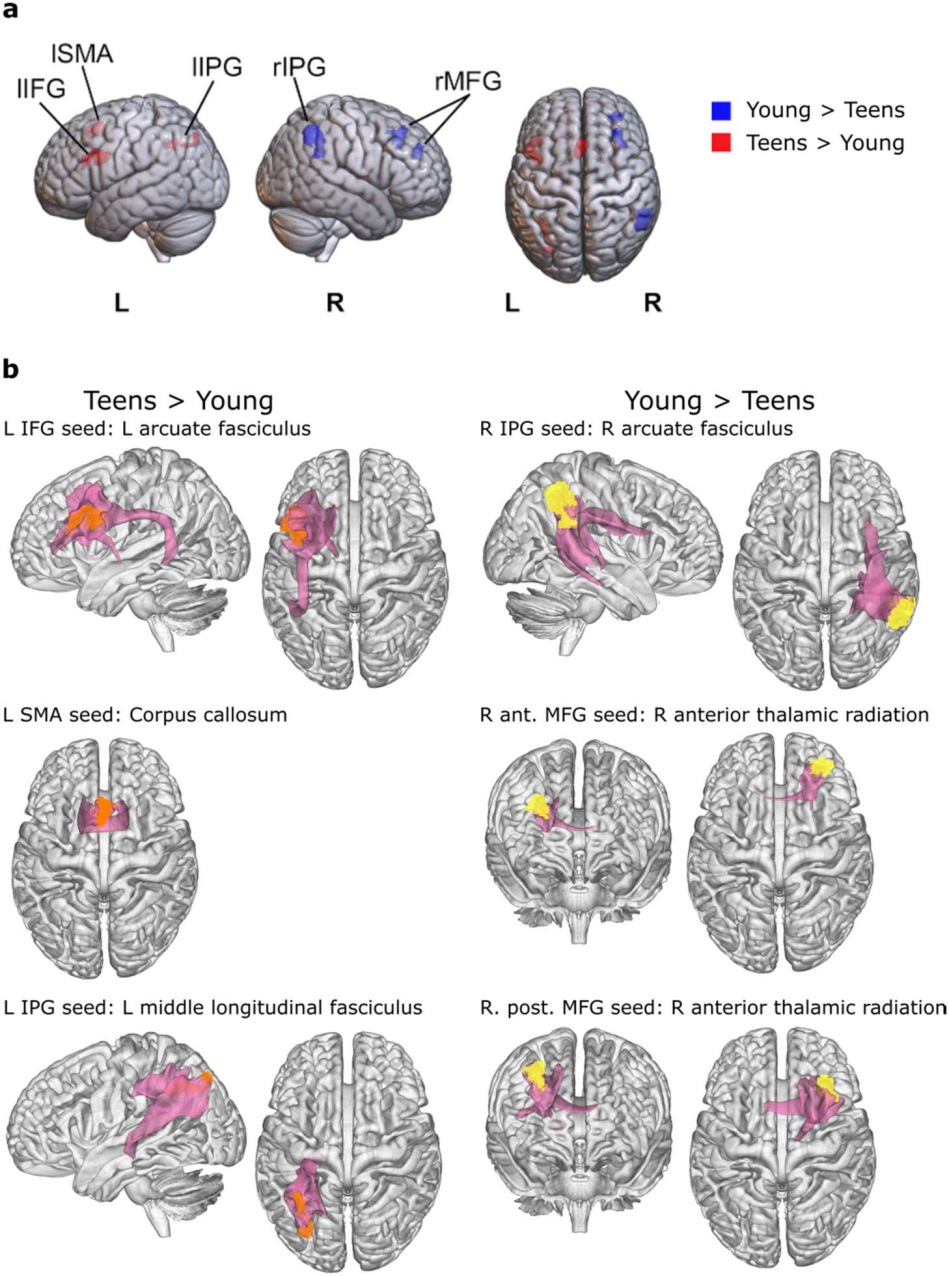
**a** Regions where fMRI activation differed between Young and Teen groups when retrieving newly learned words, adapted from Takashima et al. (2019). **b** Average visitation maps (pink tracts) for tractography seeded in the regions shown in **a**. Orange regions are the seed regions where the Teen group showed stronger activation than the Young group in the fMRI study. The yellow regions are the seed regions where the Young group showed stronger activation than the Teen group in the fMRI study. lIFG: left inferior frontal gyrus, lSMA: left supplementary motor area, lIPG: left inferior parietal gyrus, rIPG: right inferior parietal gyrus, rMFG: right middle frontal gyrus, L: left, R: right

We seeded probabilistic tractography in regions where the Young and the Teen groups exhibited differences in functional activation in response to a lexical decision task of newly learned L2 words (Takashima et al. 2019). These were the left IFG, left IPL, left SMA, two regions in the right MFG and the right IPL (Fig. 3A). When seeding from the left IFG and the right IPL, tractograms resulted in areas that include the left and right arcuate fascicle/superior longitudinal fasciculus III (AF/SLF III), respectively. The left IPL seed resulted in a tractogram that includes the middle longitudinal fasciculus. The left SMA seed resulted in a tractogram made up of corpus callosum streamlines. The right anterior and posterior MFG seeds resulted in tractograms that include streamlines running along the corpus callosum and anterior thalamic radiation (Fig. 3B).

### Age-group difference

**Fig. 4:**
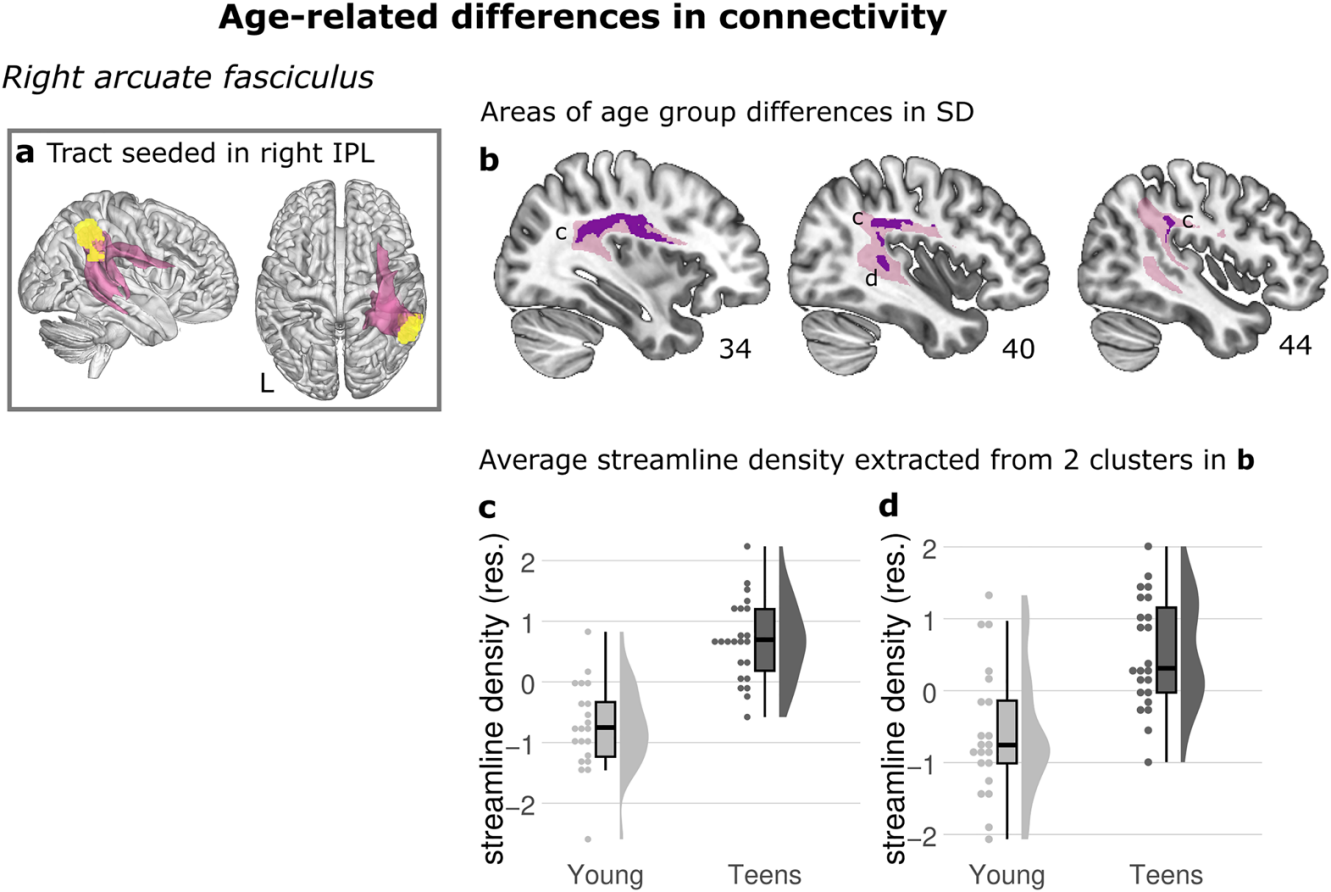
Age-related differences in connectivity in the right arcuate fasciculus. **a** Seed (yellow) and resulting average visitation map (pink) rendered in 3D of tractography seeded in the right inferior parietal lobe (rIPL) where the Young showed more BOLD activity than the Teen group in Takashima et al. (2019). **b** The purple clusters visualize a significant difference in streamline density (SD) between the Young and Teen groups in the right arcuate fasciculus (AF). The average group visitation map (pink) served as the statistical search space for group differences. **c**, **d** Average streamline density extracted from the two clusters shown in b where group differences were significant. The streamline density values plotted (y-axis) are the streamline density values averaged across all the voxels within the cluster where the significant effect was located. Standardized residuals are plotted in **c**, **d**. The results in **b** are corrected for multiple comparisons using threshold-free cluster enhancement (TFCE)

The first question that we asked was whether we could observe age group differences between the Young and the Teen groups in structural connectivity in tracts that were seeded from gray matter regions that showed functional activation differences between the groups. This group analysis revealed differences between the Young and the Teen groups in streamline density within one of the tractograms originating from the functional seeds. The Teen group exhibited higher streamline density than the Young group in the temporal-parietal section of the right arcuate fasciculus (AF), Fig. 4 and Table 2. Additionally, there was a significant difference in the corpus callosum, originating from the functional seed in the left SMA (Figure S1A). We also found an interaction of age group and L2 word learning success on streamline density in the same area (Fig. 6D).

**Fig. 5:**
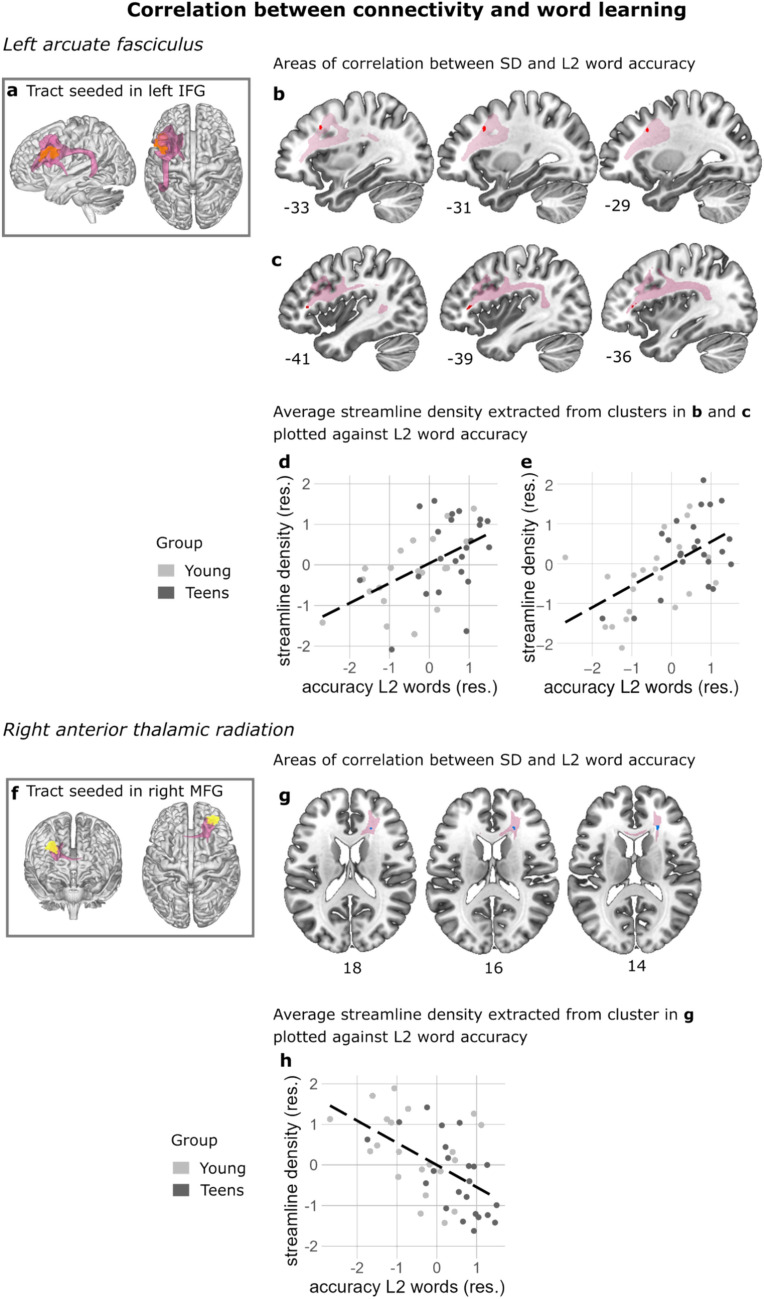
Correlation between white matter connectivity and word learning in the left arcuate fasciculus (AF) and the right anterior thalamic radiation (ATR). **a** Seed (orange) and resulting average visitation map (pink) rendered in 3D of tractography seeded in the left inferior frontal gyrus (lIFG) where the Teen group showed more BOLD activity than the Young group in Takashima et al. (2019). **b**, **c** The red clusters show areas of positive correlation between streamline density (SD) and word learning across the entire age range in the left AF. The average group visitation map (pink) served as the statistical search space for the correlation. **d**, **e** Average streamline density extracted from the two clusters shown in **b** and **c** where the positive correlation between streamline density and word learning was significant. Plot **d** visualizes the values extracted from the cluster in **b**; plot **e** visualizes the values extracted from the cluster in **c**. **f** Seed (yellow) and resulting average visitation map (pink) of tractography seeded in the right middle frontal gyrus (MFG) where the Young group showed more BOLD activity than the Teen group. **g** The blue cluster shows the area of negative correlation between streamline density and word learning across the entire age range in the right ATR. **h** Average streamline density extracted from the cluster shown in **g** where the negative correlation was significant. The scatterplots **d**, **e**, **h** are for visualization purposes only. The streamline density values plotted (y-axis) are the streamline density values averaged across all the voxels within the cluster where the significant effect was located. Standardized residuals are plotted in **d**, **e** and **h**. The results in **b**, **c** and **g** are corrected for multiple comparisons using a cluster size correction

**Table 2:** MNI coordinates, cluster size and t-value of significant effects

Seed	Main effect: age				Interaction between age and L2 word learning success				Behavioral correlation: L2 word learning success*			
	Cluster size	MNI coordinates (max)	White matter structure	*t*-value	Cluster size	MNI coordinates (max)	White matter structure	*t*-value	Cluster size	MNI coordinates (max)	White matter structure	*t*-value
Left IFG					56	− 42, − 2, 21	L SLF	4.661	76	− 29, 17, 34	L AF	1.833
									65	− 40, 32, 6	L AF	2.069
Left SMA	99	− 3, 6, 25	Corpus callosum**		1329	− 15, 7, 32	Corpus callosum	3.451				
	95	− 10, 13, 48			230	14, 14, 26		1.587				
	56	− 17, 8, 37										
Right IPL	4768	36, − 41, 27	R SLF	4.327								
	94	40, − 36, 7	R SLF	5.829								
Right MFG									54	23, 28, 16	R ATR	2.377

*ATR* anterior thalamic radiation, *SLF* superior longitudinal fasciculus, *ILF* inferior longitudinal fasciculus; *IFOF* inferior fronto-occipital fasciculus, *R* right, *L* left. *Behavioral correlation effects are cluster size corrected, all other findings are TFCE corrected. For TFCE corrected results, clusters larger than 10 voxels are reported. **The three clusters in the corpus callosum that exhibit a main effect of age group overlap to a large extent with the corpus callosum findings from the interaction analysis.

### Behavioral correlation with memory for newly learned L2 words

Next, we correlated streamline density with L2 word learning success across the two age groups. Here we wanted to determine whether there were common regions where structural connectivity related to L2 word learning success across the two age groups. Streamline density correlated positively with L2 word learning success in the anterior part of the left arcuate fasciculus (AF) as well as more medial white matter of the AF. In both regions, better memory for newly learned words was related to higher streamline density. Additionally, streamline density correlated negatively with L2 word learning success in the right anterior thalamic radiation. Here better memory for newly learned words was associated with lower streamline density, Fig. 5 and Table 2. We repeated the analysis to include L1 vocabulary and digit span as covariates. In this analysis, the cluster in Fig. 5C is no longer significant, for an overview of the results that include these covariates, see Table S1.

**Fig. 6:**
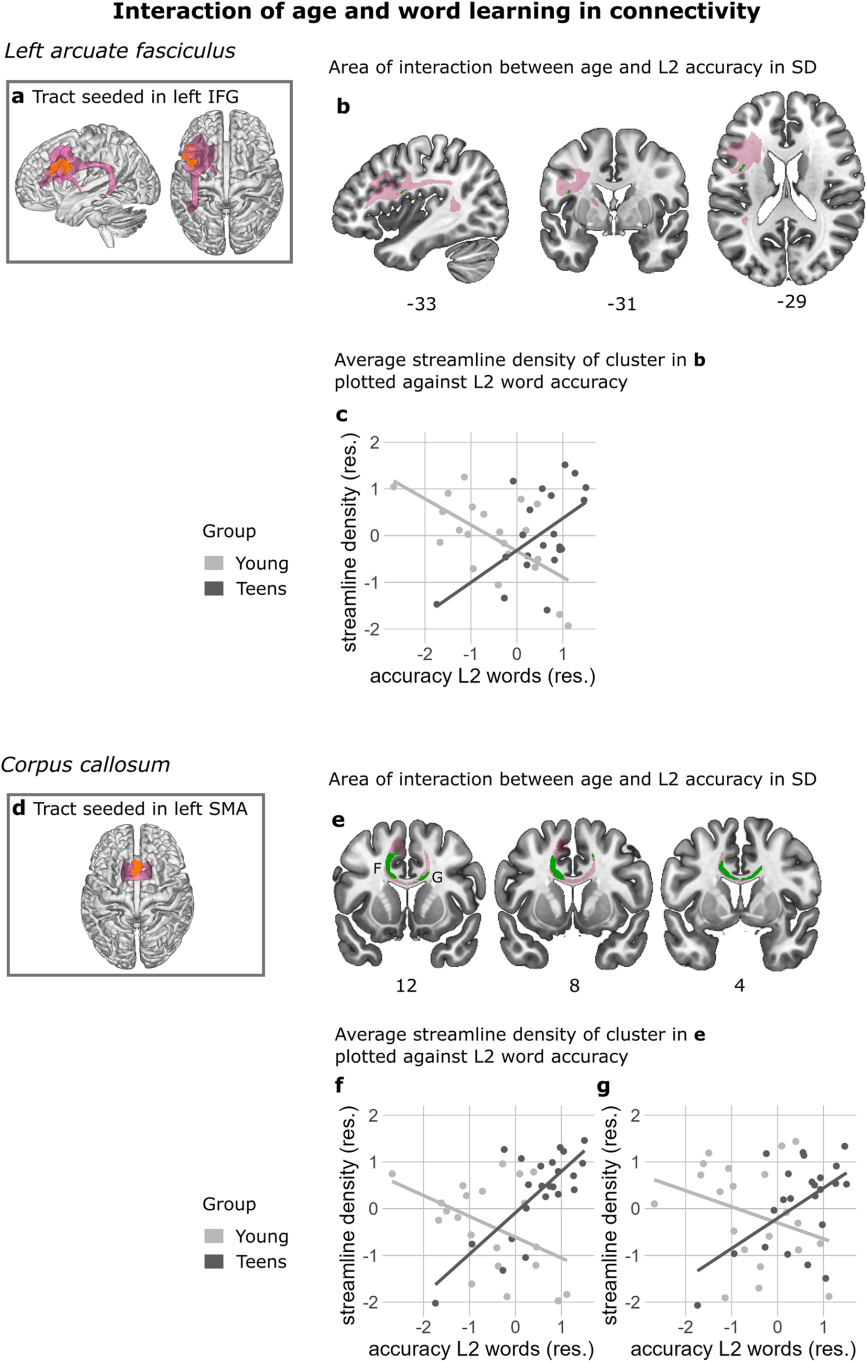
Interaction of age group and word learning on white matter connectivity in the left arcuate fasciculus (AF) and the corpus callosum. **a**, **d** Seeds (orange) and resulting average visitation maps (pink) rendered in 3D of tractography seeded in the left inferior frontal gyrus (IFG) **a** and the left SMA **d** where the Teen group showed more BOLD activity than the Young group in Takashima et al. (2019). **b**, **e** The green clusters show areas of interaction where the Young group exhibited a negative correlation between streamline density (SD) and word learning, whereas the Teen group showed the opposite effect in the left AF (**b**) and the corpus callosum (**e**). The average group visitation map (pink) served as the statistical search space for the interaction effect. **c**, **f**, **g** Average SD extracted from one cluster in the left AF shown in (**b**) and two clusters in the corpus callosum shown in **e** where the interaction was significant. The scatterplots **c**, **f**, **g** are for visualization purposes only. The SD values plotted (y-axis) are the SD values averaged across all the voxels within the cluster where the significant effect was located. Standardized residuals are plotted in **c**, **f** and **g**. Only clusters larger than 10 voxels are plotted. The results in **b**, **e** are corrected for multiple comparisons using threshold-free cluster enhancement (TFCE)

### Interaction between age group and memory for newly learned L2 words

The age range in this sample was not continuous, with the Young group between the ages of 8 and 10 years and the Teen group between 14 and 16 years. For this reason, we conducted an interaction analysis to follow up on the correlation analysis conducted across the whole sample, described above. The interaction analysis was done to verify that the correlation was not a spurious effect between behavior and streamline density due to the discontinuous age range covered in our sample. We found interaction effects of age group (Young or Teen) and L2 word learning success on streamline density in the left AF as well as the corpus callosum (Fig. 6 and Table 2). The pattern of interaction was the same in both the left AF and the corpus callosum: the Teen group exhibited a positive correlation indicating that better memory for newly learned L2 words was related to higher streamline density; the Young group showed a negative correlation, i.e. better memory for newly learned words was related to lower streamline density. 

No group, correlation or interaction effects were found in streamline density in the tracts emanating from the left inferior parietal or the right posterior MFG seeds (Fig. 3).

### Regression analyses: Japanese word learning success in relation to age and white matter

Behaviorally, a strong positive relationship existed between age and L2 word learning success (*r*(42) = 0.55, *p* < 0.001): the regression analysis showed that age explained 30.0% of the variation of the L2 word learning success across all participants, R^2^ = 0.30, F(1, 42) = 17.99, *p* = 0.0001, see Table 3. Furthermore, we wanted to determine if the age group effect in the right AF (Sect. “Age-group difference”) could also be linked to behavioral performance. L2 word learning success correlated significantly with the average streamline density extracted from the two clusters that showed significant age effects located in the temporal-parietal part of the right AF (Fig. 4), the largest cluster (*r*(42) = 0.30, *p* = 0.046) and the second cluster (*r*(42) = 0.49, *p* = 0.001). An additional both-ways stepwise regression with the streamline density from the two significant group effect clusters entered as predictors indicated that the model including only the streamline density of the second cluster (Fig. 4D) as predictor was the best fitting one, significantly explaining 23.6% of the variance in L2 word learning success, R^2^ = 0.236, F(1, 42) = 13.0, *p* = 0.0008, see Table 3. Follow-up mediation analyses revealed no indirect effect of age on L2 word learning success through white matter differences within the largest cluster (*t(42)* = 1.67, *p* = 0.103) or second cluster (*t (42)* = −1.10, *p* = 0.278). Indirect effect = −0.797, SE = 0.716, 95% CI [−2.289, 0.521]; second cluster: Indirect effect = 0.774, SE = 0.635, 95% CI [-0.192, 2.289].

**Table 3:** Regression model outputs of analyses described in Sect. “Regression analyses: Japanese word learning success in relation to age and white matter”

Model	Predictors	Estimate (std. error)	*t*-value	*p*
L2 word learning success ~ age	Age	2.881 (0.679)	4.242	0.0001
L2 word learning success ~ SD cluster (small)	SD cluster small	81.84 (22.70)	3.61	0.0008

*SD* streamline density

## Discussion

In this study we set out to examine the structure–function relationship of age-related differences in second language (L2) word learning in a developmental sample. We investigated the white matter tracts underlying brain regions that showed age-related functional activation differences when recognizing newly learned L2 words (Takashima et al. 2019). We found group differences in white matter connectivity in the right arcuate fasciculus (AF); here, the Teen group showed stronger connectivity than the Young group. Furthermore, we found correlations between white matter connectivity and memory for L2 words, across the two age groups: the relationship was positive in the left AF and negative in the right anterior thalamic radiation (ATR) which indicates that in both the Young and the Teen group, higher connectivity in the left AF is related to better memory for L2 words, whereas in the right ATR, lower connectivity is linked to better memory for L2 words. The novelty of the present study lies in the investigation of structural connections of the previously reported functional age differences in L2 word learning (Takashima et al. 2019).

Additionally, we wanted to determine if the group effect in the right AF could also be linked to behavioral performance. To this end, we correlated the streamline density in the two clusters of the right AF with memory for L2 words. This analysis revealed a correlation between streamline density and word learning in the right AF. The pattern of effects of positive and negative correlation, as well as the different direction of effects for the Young and the Teen groups underlines the dynamically changing pattern of white matter connections that play a role in L2 word learning.

In addition to our main findings, we report an interaction between memory for newly learned L2 words and age group in the white matter connectivity in the left AF as well as the corpus callosum. In both tracts, while the Teen group showed a positive correlation between memory for L2 words and connectivity, the opposite pattern was observed in the Young group, thus whereas higher streamline density was related to higher accuracy on memory for L2 words in the Teen group, lower streamline density was related to higher accuracy on memory for L2 words in the Young group.

### Arcuate fasciculus/ superior longitudinal fasciculus III

Compared to the Young group, the Teen group exhibited stronger connectivity in the right arcuate fasciculus/ superior longitudinal fasciculus III (AF/SLF), seeding from the right inferior parietal lobe (IPL). Furthermore, the connectivity strength of these areas that showed differences between groups was positively correlated with L2 word learning ability. This indicates that individuals with stronger connectivity in the right AF are better at L2 word learning than individuals with weaker connectivity. In addition to this group difference, we also found that across our whole sample, connectivity in sections of the left AF correlated positively with L2 learning ability. The AF connects the posterior part of the superior temporal gyrus and the anterior part of the inferior parietal gyrus with the ventral premotor and the inferior frontal gyrus (Schmahmann et al. 2009). The AF and the cortical regions it connects, albeit in the left hemisphere, has repeatedly been linked to word learning, specifically in adults (Lopez-Barroso et al. 2013; Hofstetter et al. 2017) where it has also been related to L2 learning (Wei et al. 2024). In children, vocabulary growth between the ages of 4 and 10 years has been related to the white matter microstructure of the left AF at age 14 (Su et al. 2018).

Furthermore, a recent study investigating word learning in children between the ages of 10 and 12 years found that memory for words directly after learning as well as after a nap was positively correlated with FA in the bilateral arcuate and the right uncinate fasciculus (Van Rijn et al. 2023). Our findings in the right AF are therefore consistent with those reported by van Rijn and colleagues (2023). The age-related differences observed within the right AF could be explained by increased white matter maturation in the Teen compared to the Young group.

Interestingly, we did not find a similar age-related difference in connectivity between our groups in the left AF. This could be explained in several ways: first, the connectivity is already relatively strong in both children and adolescents, suggesting faster maturation of the left compared to the right AF. A second explanation is that the adolescents still have lower connectivity in the left AF as well, suggesting slower maturation of the left AF compared to the right. Both scenarios would suggest that the maturation of the AF is lateralized. One study which investigated lateralization in participants between 0 and 28 years found rightward lateralization in raw data, but leftward lateralization in normalized data (Wilkinson et al. 2017). For both, no age-effect of lateralization was detected. With the results from the current study, we cannot tease apart the underlying reason for the reported pattern of effects, since the absence of an age-related effect in one hemisphere does not rule out that an age-related effect exists. This would need to be tested directly, in a lateralization analysis, which was not the goal of the present study.

By now, the protracted maturation of white matter tracts is widely accepted, and the development of the AF appears to peak relatively late, in the late 20s on average (Zhang et al. 2007; Verhoeven et al. 2010; Paus 2010; Lebel et al. 2012). This is in line with a previous study that found higher connectivity in both the left and right AF for adults compared to 7-year olds, which demonstrated that children have a less matured language network compared to adults (Brauer et al. 2011). The results from the current study indicate that this maturation difference for children can already be observed in comparison to teens. In a future study, it would be interesting to include an adult group to compare to, as this would provide insight about when the neural architecture supporting word learning is established, that is, whether the Teen group recruits the same brain structures as adults.

### Right anterior thalamic radiation

When correlating streamline density with L2 word learning ability in the whole group, we found a negative correlation in the right anterior thalamic radiation (ATR). The ATR is thought to connect the thalamus (i.e., medial nuclear complex) with the dorsolateral prefrontal cortex (Kahle and Frotscher 1976; Coenen et al. 2012). This tract is essential for executive functioning (Mamiya et al. 2018) and declarative memory and thus learning (Van Der Werf et al. 2003; Leszczyński and Staudigl 2016). So far, only a small number of studies have linked white matter microstructure in the ATR to L2 experience or L2 learning and only in adults (Cummine and Boliek 2013; Kuhl et al. 2016; Rossi et al. 2017). For example, while one study linked higher FA values with L2 proficiency in the left ATR in adults (Rossi et al. 2017), two studies observed decreased FA values within the left or bilateral ATR for adult bilinguals when compared to monolinguals (Cummine and Boliek 2013; Kuhl et al. 2016). Although in our study we expected connectivity to show the opposite pattern of correlation, that is, a positive correlation with L2 word learning abilities, our finding that L2 learning success was negatively associated with the streamline density within the rATR is also supported by previous findings. While other studies besides the two mentioned above have also reported negative correlations between white matter structure and language-related abilities (e.g. Madhavan et al. 2014; Jung et al. 2019), it is unclear what gives rise to these negative correlations.

### Interaction with age group and link between connectivity and word learning

We tested for interaction effects of age and L2 word learning ability on streamline density. We conducted this analysis because we wanted to ensure that the correlation that we carried out between L2 word learning ability and streamline density was not a spurious effect, since the ages covered by our sample are not continuous. In the left AF and the corpus callosum, we found an interaction effect. In both tracts, we detected the same pattern of effects, that is, for the Young group between the ages of 8 and 10, we observed a negative correlation, with those who exhibited lower streamline density having a better L2 word learning ability, whereas in the Teen group the pattern was reversed: higher streamline density was related to better L2 word learning ability. As discussed above, the AF, and in particular the left AF has been shown in multiple studies to be related to word learning in both adults and children. The corpus callosum connects the two hemispheres and has been linked in children to vocabulary, verbal fluency and verbal span (Bartha-Doering et al. 2018). While this pattern of effects may reflect an interesting developmental discontinuity, the results should be treated as initial indications of such discontinuity until the effect can be replicated in a larger sample.

### Linking function and structure

In this sample, the left IFG, part of the PFC, showed more activation in Teens than in the Young group when retrieving newly learned L2 words (Takashima et al. 2019). The current analysis of the structural connectivity data reveals that the connectivity of the left IFG, though it did not differ between groups, is positively related to L2 memory for words. That is, participants with stronger connectivity of the left PFC exhibited better memory for words. This is in line with the two-component framework of memory development, which posits that the late development of the PFC plays a role in memory improvement that has been observed across childhood (Shing et al. 2010). The results in the current study show that in addition to the age-related differences in functional activation of the left PFC reported by Takashima et al. (2019), stronger connectivity of this region is also related to better memory (word learning) performance. What is puzzling at first glance is that the Teen group has stronger connectivity in the right AF than the Young group, since in the seed region, the right IPL, the Young group showed more activation in response to newly learned L2 words than the Teen group (Takashima et al. 2019). One possible explanation for this mismatch is that while the functional result reflects the relative immaturity of the Young group, namely that the right hemisphere structures are additionally recruited by the Young group to complete the task, the structural result reflects the expected pattern of maturation of the white matter.

### Connectivity strength in development

White matter, consisting of structural connections between brain regions, matures slowly and this trajectory influences cognitive abilities throughout development (Johansen-Berg 2010). While connectivity strength measured by streamline density has been investigated in children previously (Grosse Wiesmann et al. 2017; Qi et al. 2019; Kuhl et al. 2020), it has not been linked to word learning in development. Previous studies investigating white matter in relation to word learning in development have focused on diffusion tensor indices such as fractional anisotropy (FA). Thus, the current findings of age differences in connectivity strength underlying age differences in functional activity as well as the correlation between connectivity strength and L2 word learning success extend our understanding of the role of white matter connectivity strength in the development of L2 word learning.

### Limitations

Several limitations of the current study should be mentioned. One limitation of our study sample is that we did not include an adult group, rather our data collection focused on children around the age of puberty. Future studies should additionally include an adult group to conclude whether children and teens have structural and functional differences compared to adults. An additional limitation is the relatively small sample size, although this is not uncommon for developmental neuroimaging studies. Nevertheless, the small sample size could have reduced the power to detect true effects. Therefore, it is possible that age-group differences exist in the other white matter tracts that were included in the analysis of the paper. Furthermore, we may have been unable to detect additional regions where structural connectivity correlates with word learning ability due to low power. The generalizability of our findings may also be limited by the small sample size. Ideally, an analysis of a large longitudinal sample with age continuously sampled covering childhood into adulthood would give the clearest picture on how and when second language word learning changes with age (Menks et al. 2022, 2024). Finally, white matter tractography is associated with several methological difficulties. One of these is the crossing fiber problem. When a single voxel contains several fiber bundles, this causes difficulty for the reconstruction of the tract. However, we used probabilistic tractography instead of deterministic tractography in the current study, and this alleviates the crossing fiber problem to a certain extent (Behrens et al 2007). Notwithstanding the limitations of this study, the results provide empirical evidence that the age group difference in functional activation reported in our previous study (Takashima et al. 2019) is accompanied by age group differences in white matter connectivity in the right arcuate fasciculus, and that the structure of this tract is also related to L2 word learning ability.

## Conclusion

In this study, we investigated the structural connectivity underlying age-related differences in functional activation related to word learning. The previously published fMRI results from the same sample showed that the Teen group recruited regions in the left hemisphere more than the Young group, whereas the Young group had more activation in the right hemisphere (Takashima et al. 2019). In the white matter connectivity of these regions, we found stronger connectivity in the right AF in the Teen group compared to the Young group and that participants with stronger connectivity were better at word learning. In the connectivity of the left IFG, namely the left AF, we also found that stronger connectivity was related to better word learning. Since stronger connectivity is likely in large part related to maturation, this finding is in line with previous studies that report better episodic memory in adults than children related to the maturation of the prefrontal cortex. Our findings thus suggest that also the maturation of the structural connectivity of the prefrontal cortex contributes to the improvement in episodic memory in the service of word learning. Future research should probe the neural mechanisms underlying L2 word learning in a continuous age group, ideally using a longitudinal design, to shed more detailed light on the developmental trajectory of this process.

To study structural connectivity at different ages across development allows to link maturational brain processes to functional and behavioral changes in word learning. Therefore, our findings can be interpreted in the light of dynamic systems theory which provides a framework to study principles and fundamental mechanisms of change in second language development (e.g. Han et al. 2023). Furthermore, the findings add a so far understudied part, namely changes in brain structure during childhood and adolescents, to the complex system of interacting components as defined in the framework of the dynamics systems theory.

## Supplementary Information

Below is the link to the electronic supplementary material.

Supplementary file1 (DOCX 343 KB)

## Data Availability

The preprocessed data and code used for data analysis will be available on the Radboud Data Repository: 10.34973/fwbx-f057.
