# Supplementary material for: White matter connectivity linked to novel word learning in children
Source: Brain Struct Funct. 2024 Sep 26;229(9):2461–77. doi: 10.1007/s00429-024-02857-6 (PMC11612013; doi:10.1007/s00429-024-02857-6)
Supplement: Supplementary file 1 — Supplementary file1 (DOCX 343 KB) [file 429_2024_2857_MOESM1_ESM.docx]

**Supplemental Materials**

Supplemental Figures


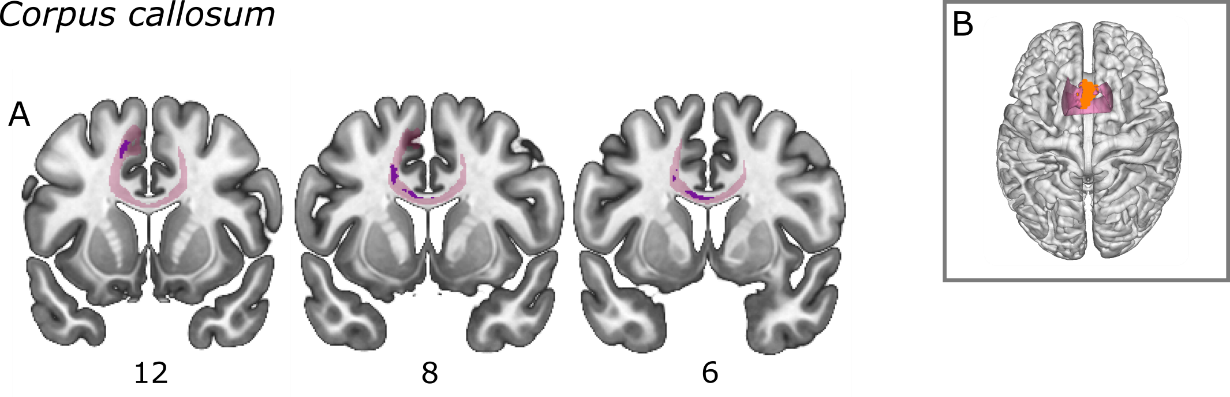


**Supplemental Figure 1** Age-related differences in connectivity in the corpus callosum. A) Difference in streamline density (purple) between children and teens in the right arcuate fasciculus, the average group visitation map (pink) served as the statistical search space for group differences. B) Seed (yellow) and resulting group visitation map (pink) rendered in 3D of tractography seeded in the right inferior parietal lobe where children showed more BOLD activity than teens in Takashima et al. (2019).

Supplemental Tables

| Seed | **Main Effect: Age** | | | **Interaction between age and L2 word learning success** | | | **Behavioral Correlation: L2 word learning success*** | | |
| --- | --- | --- | --- | --- | --- | --- | --- | --- | --- |
|  | Cluster size | MNI coordinates (Max) | White matter structure | Cluster size | MNI coordinates (Max) | White matter structure | Cluster size | MNI coordinates (Max) | White matter structure |
| Left IFG |  |  |  | 75 (not TFCE) | -39, 5, 18 | L SLF | 73 | -29, 17, 34 | L AF |
| Left SMA | 498 | -17, 8, 37 | corpus callosum** | 945 | -15, 7, 32 | corpus callosum |  |  |  |
|  |  |  |  | 38 | 13, 3, 29 |  |  |  |  |
| Right IP | 4208 | 36, -41, 27 | R SLF |  |  |  |  |  |  |
| Right MFG |  |  |  |  |  |  | 69 | 25, 29, 13 | R ATR |

**Supplemental Table 1** MNI coordinates and cluster size of significant effects when including L1 vocabulary and digit span scores as additional covariates. ATR= anterior thalamic radiation, SLF = superior longitudinal fasciculus, ILF = inferior longitudinal fasciculus; IFOF = inferior fronto-occipital fasciculus; R = right; L = left. *Behavioral correlation effects are cluster size corrected, all other findings except for the cluster in the left IFG that shows an interaction are TFCE corrected. For TFCE corrected results, clusters larger than 10 voxels are reported. **The cluster in the corpus callosum that exhibits a main effect of age group overlaps to a large extent with the corpus callosum findings from the interaction analysis.
